# Semi-automatic synthesis and biodistribution of *N*-(2-^18^F-fluoropropionyl)-bis(zinc (II)-dipicolylamine) (^18^F-FP-DPAZn2) for AD model imaging

**DOI:** 10.1186/s12880-017-0200-1

**Published:** 2017-04-21

**Authors:** Fuhua Wen, Dahong Nie, Kongzhen Hu, Ganghua Tang, Shaobo Yao, Caihua Tang

**Affiliations:** Department of Nuclear Medicine, The First Affiliated Hospital, Sun Yat-Sen University, Guangzhou, 510080 China

**Keywords:** ^18^F-NFP, ^18^F-FP-DPAZn2, Semi-automatic synthesis, Zinc(II)-dipicolylamine, Cell death

## Abstract

**Background:**

Phosphatidylserine (PS)-targeting positron emission tomography (PET) imaging with labeled small-molecule tracer is a crucial non-invasive molecule imaging method of apoptosis. In this study, semi-automatic radiosynthesis and biodistribution of *N*-(2-^18^F-fluoropropionyl)-bis(zinc(II)-dipicolylamine) (^18^F-FP-DPAZn2), as a potential small-molecule tracer for PET imaging of cell death in Alzheimer’s disease (AD) model, were performed.

**Methods:**

^18^F-FP-DPAZn2 was synthesized on the modified PET-MF-2V-IT-I synthesizer. Biodistribution was determined in normal mice and PET images of AD model were obtained on a micro PET-CT scanner.

**Results:**

With the modified synthesizer, the total decay-corrected radiochemical yield of ^18^F-FP-DPAZn2 was 35 ± 6% (*n* = 5) from ^18^F^−^ within 105 ± 10 min. Biodistribution results showed that kidney has the highest uptake of ^18^F-FP-DPAZn2. The uptake of radioactivity in brain kept at a relatively low level during the whole observed time. In vivo ^18^F-FP-DPAZn2 PET images demonstrated more accumulation of radioactivity in the brain of AD model mice than that in the brain of normal mice.

**Conclusions:**

The semi-automatic synthetic method provides a slightly higher radiochemical yield and shorter whole synthesis time of ^18^F-FP-DPAZn2 than the manual operation method. This improved method can give enough radioactivity and high radiochemical purity of ^18^F-FP-DPAZn2 for in vivo PET imaging. The results show that ^18^F-FP-DPAZn2 seems to be a potential cell death tracer for AD imaging.

## Background

Programmed cell death acts a vital physiological and pathological role in the biological process. Many pathological conditions, such as cancer, cardiovascular diseases, neurodegenerative disorders, auto-immune diseases, are associated with cell death [1]. β-amyloid (Aβ) is a main etiologic agent in AD [2], consisting of Aβ (1–40) and Aβ (1–42) peptides in the AD brain. Aβ is expected to be an important target of presymptomatic diagnosis and treatment of AD [3, 4]. In the AD brain, Aβ accumulation is to increase nitric oxide production, cytochrome c release into the cytoplasm and oxidative phosphorylation, leading to apoptosis or cell death [5, 6]. Noninvasive, functional, and molecular imaging of cell death may be of great value in the future clinical practice for disease diagnosis and treatment evaluation [7, 8].

Cell death can be determined by labeled Annexin V based on the recognition of extracellular phosphatidylserine (PS) [9]. Monitoring the cell surface expression of PS is used in detection of an early stage apoptosis and necrosis [10–12]. Fluorine-18 labelled annexin V as a positron emission tomography (PET) tracer can be used for apoptosis imaging. However, labeled Annexin V showed unfavourable pharmacokinetics characteristics of slow clearance from blood because of relatively large labelled protein [13, 14]. DPAZn2 fluorescent probes can differentiate the dead and the dying cells from the normal cells and selectively bind PS of bacteria in heterogeneous biological medium [15–18]. It is well-known that synthetic DPAZn2 complexes can be selectively recognized PS-rich membranes and act as molecular imaging probes for cell death. But, there is a necessary to improve in vivo imaging performance by selectively increasing target affinity and to decrease off-target accumulation [19]. Thus, 4-^18^F-fluorobenzoyl-bis(zinc(II)-dipicolylamine (^18^F-FB-DPAZn2) was developed as a new DPAZn2 tracer for PET imaging of tumor-treating animal models [20]. However, ^18^F-FB-DPAZn2 with high uptake in liver and bowel was not good for abdominal PET imaging.

In the previous study, we reported the synthesis of ^18^F-FP-DPAZn2 probe [21], which had smaller molecular weight than ^18^F-FB-DPAZn2. The biodistribution demonstrated that there was lower uptake of ^18^F-FP-DPAZn2 than that of ^18^F-FB-DPAZn2 in the abdomen, because ^18^F-NFP possessed better characteristics compared with *N*-succinimidyl-4-^18^F-fluorobenzoate (^18^F-SFB) for marking small-molecule peptides and peptide hormones [22]. Also, ^18^F-NFP had relatively higher in vivo metabolic stability and weaker hydrophobicity than ^18^F-SFB [23]. Furthermore, ^18^F-NFP was an ^18^F-radiolabeling prosthetic group with small-molecule weight for labeling peptides and had less influence on the biologic characteristics of peptides [24]. Therefore, we first labelled DPAZn2 with ^18^F-NFP to obtain ^18^F-FP-DPAZn2 by manual operation. But the manual operation was time-consuming and radiochemical yield was low, especially, operators could accept an excess of radiation dose, which urged us to develop a semi-automatic synthetic procedure or an automatic synthetic protocol of ^18^F-FP-DPAZn2.

In this study, we successfully performed semi-automatic synthesis of ^18^F-FP-DPAZn2 using the modified synthesizer, which gave a slightly higher radiochemical yield and shorter synthesis time than the manual method. Additionally, in vivo biodistribution of ^18^F-FP-DPAZn2 was determined and first PET imaging of cell death in double transgenic AD models with ^18^F-FP-DPAZn2 was also investigated.

## Methods

### Materials

All chemical reagents obtained commercially with analytical grade and used without any purification. QMA Sep-Pak cartridges were purchased from Waters. The cartridges pretreated with 8.4% NaHCO_3_ and water. Reversed-phase Sep-Pak C_18_ plus cartridges were pretreated with ethanol and water, and Oasis HLB cartridges were pretreated with the same method. PET-MF-2V-IT-I synthesizer with a built-in RP-HPLC system was purchased from Beijing PET Co. (Beijing. China). The HPLC system equipped with a semi-preparation RP-C18 column (10 × 250 mm). Syringe filters with diameter 13 mm and sterilizing filters with pore size 0.22 μm were purchased from Nalge Nuc International.

### Synthesis of the precursor DPA2

The precursor DPA2 was prepared by the reported procedure [25], with slight modifications [20, 26]. In brief, according to the improved method [20, 26], after 3,5-bis-hydroxymethyl phenol reacted with *N*-Boc-{2-[2-(2-p-toluenesulfonyl-ethoxy)-ethoxy]-ethyl}monoamine, the two hydroxyl groups of reaction product were halogenated with methanesulfonyl chloride instead of CBr_4_ [25], and then reacted with 2,2′-dipicolylamine and deprotected with trifluoroacetic acid (TFA) to obtain the precursor DPA2. The total chemical yield was about 5.0%.

### Automated synthesis of ^18^F-NFP

**Fig. 1:**
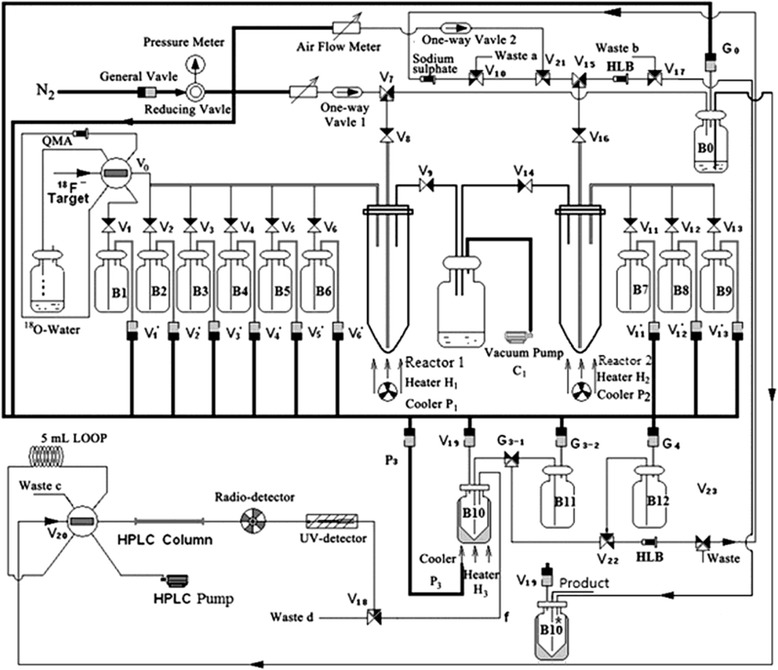
Schematic diagram of PET-MF-2V-IT-1 synthesis module

**Fig. 2:**
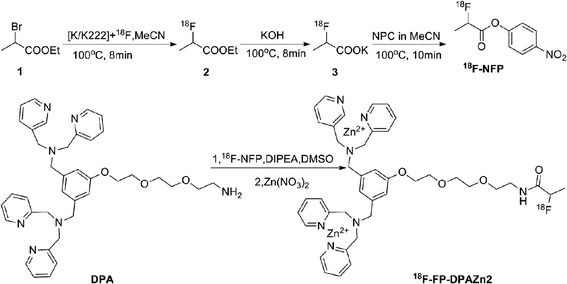
The radiosynthesis route of ^18^F-NFP and ^18^F-FP-DPAZn2

^18^F-F^−^ was obtained from the cyclotron (IBA Technologies). ^18^F-NFP was synthesized using three-step one-pot procedure on the improved synthesis module as shown in Fig. 1. The radiosynthetic route of ^18^F-NFP described by Hu [22] was shown in Fig. 2. A solution of 18-Crown-6 (K_222_) (15 mg) and K_2_CO_3_ (3 mg) in 0.9 mL acetonitrile and 0.1 mL water was kept in vial B1. Anhydrous acetonitrile (2 mL) was kept in vial B2 and 5 mg of ethyl-2-bromopropionate was dissolved in 1 mL anhydrous acetonitrile (vial B3). Potassium hydroxide aqueous solution (0.2 M, 0.2 mL) was added in vial B4 and 40 mg of bis(4-nitrophenyl) carbonate dissolved in 1 mL acetonitrile was kept in vial B5. Five percent of acetate aqueous solution (1 mL) was kept in vial B6 and 0.1% of trifluoroacetic acid aqueous solution (40 mL) was kept in vial B10. Water (1 mL) was added in vial B11 and ether was kept in vial B12.

Around 1.85 GBq (50 mCi) of ^18^F^−^ in ^18^O-H_2_O were captured by an ion exchange resin and eluted with K_222_ (vial B1) into the reactor 1. The mixture was dried by azeotropical evaporation with acetonitrile at 115 °C under a nitrogen flow. Then, the complex was dried again with anhydrous acetonitrile (vial B2). After cooling down to 40 °C, compound 1 (as shown in Fig. 2) in vial B3 was transferred into the reactor 1. The solution was kept at 100 °C for 480 s to produce compound 2. When temperature was cooled down to 40 °C, potassium hydroxide (vial B4) was added to the reactor 1 and compound 2 was hydrolyzed to compound 3 at 100 °C for 480 s. Then, bis(4-nitrophenyl) carbonate (vial B5) was transferred into the reactor 1, following the mixture reacted at 100 °C for 600 s. Acetate aqueous solution (vial B6) was added into the reactor 1, when the temperature of the reactant decreased to 40 °C. After the neutralization, the mixture solution was added to vial B0 and the mixture was separated by semi-preparation HPLC, with mixture of 0.1% TFA in water and 0.1% TFA in MeCN (55/45, v/v) as mobile phase (UV 254 nm, 4 mL/min). The purified product ^18^F-NFP was diluted by 40 mL water containing 0.1% TFA (vial B10) and the resulting solution was concentrated by Oasis HLB cartridge. The cartridge was rinsed with water (vial B11), dried with N_2_ flow, and eluted the product with ether into vial B12. The ether solution was passed through a Na_2_SO_4_ column under N_2_ flow into reactor 2. Finally, the ether was evaporated with nitrogen at 30 °C to give compound 4.

### Radiosynthesis of ^18^F-FP-DPAZn2

^18^F-FP-DPAZn2 was synthesized on the PET-MF-2V-IT-I synthesizer. The radiosynthesis route of ^18^F-FP-DPAZn2 was shown in Fig. 2. Five hundred 500 micrograms of precursor DPA2 dissolved in anhydrous DMSO (200 μL) and *N,N*-diisopropylethylamine (20 μL) were kept in vial B7, 0.5% acetic acid solution (20 mL) was kept in vial B8, and ethanol (2 mL) was added in vial B9. Fifteen mM Zn(NO_3_)_2_ aqueous solution (10 μL) was kept in vial B10*.

DPA2 solution (vial B7) was transferred into the reactor 2 and reacted with compound 4 at 40 °C for 600 s. After that, the mixture was quenched with an acetic acid solution (10 mL) (vial B8) and concentrated by passing through Oasis HLB cartridge. Then, the product ^18^F-FP-DPA2 was achieved by washing the cartridge with the remaining acetic acid in vial B8, following by eluting with ethanol (vial B9). The eluate was transferred into vial B10*, which contained 10 μL of 15 mM Zn(NO_3_)_2_ aqueous solution. Vial B10* was in the place of vial B10. The reaction took place at 70 °C for 600 s. Finally, the compound 6 was obtained by passing through Millipore filter, and then diluted with saline, to keep the alcohol content less than 10%.

### Determination of radiochemical purity

Raio-HPLC analysis was used to confirm the compound 6 identity by co-injection with the standard (^19^F-FP-DPA). Analytical conditions were identical with reference [22]. The standard (^19^F-FP-DPA) was prepared by the similar synthesis method to ^18^F-FP-DPA2 and identified by mass spectrometry.

### In vivo biodistribution

Sixteen Kunming mice were used to determine in vivo biodistribution at 10, 45, 60 and 90 min point after injection of ^18^F-FP-DPAZn2. Each mouse was injected with about 20–40 μCi of radiotracer. Four mice each group was killed in the proper order, then blood, interested organs and tissues were dissected, weighed, and ^18^F radioactivity was measured by a counter. The results were background-subtracted and decay corrected to the injected time and took the average. Data were presented as % ID/g.

### PET Imaging of double transgenic AD model

Three seven-month old double transgenic AD mice from B6C3-Tg (APPswe, PSEN1dE9) 85 Dbo/J mice were acquired from Guangdong Medical Laboratory Animal Center. Inveon micro-PET scanner (Siemens) was used for the ^18^F-FP-DPAZn2 PET-CT study. Each animal was injected with 3.7-7.4 MBq of ^18^F-FP-DPAZn2 in 100–200 μL of saline. The PET-CT scan was performed according to the reference [27].

## Results

### The semi-automatic synthesis

**Fig. 3:**
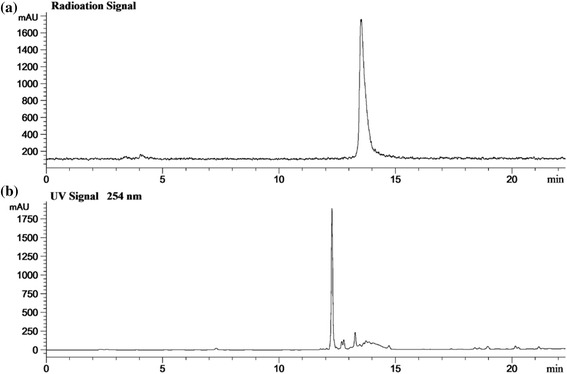
HPLC analysis of purified ^18^F-FP-DPA2 solution coinjected with the standard FP-DPA2. **a** Radioactive chromatogram; **b** UV chromatogram at 254 nm

The radiosynthesis of ^18^F-FP-DPA2 included ^18^F-NFP radiosynthesis and ^18^F-acylation reaction. The automatic synthesis of ^18^F-NFP was performed via a three-step reaction procedure. ^18^F-acylation reaction of the precursor DPA2 with ^18^F-NFP was also automatic synthesis. Finally, the chelation reaction of ^18^F-FP-DPA2 with Zn^2+^ gave the final product ^18^F-FP-DPAZn2. The total corrected radiochemical yield of ^18^F-FP-DPAZn2 was 35 ± 6% (*n* = 5) from ^18^F^−^ with 105 ± 10 min. The specific activity was more than 519 MBq/μmol. The radiochemical purity of ^18^F-FP-DPAZn2 was greater than 99% base on radio-HPLC (Fig. 3). The radioactive product was identified using HPLC with co-injection of ^18^F-FP-DPA2 and non-radioactive standard FP-DPA2 at 254 nm (UV) (as shown in Fig. 3). Figure 3 revealed that the retention time of ^18^F-FP-DPA2 in radioactive chromatogram (a) was the same as that of the standard FP-DPA2 in the UV chromatogram (b). FP-DPA2 ESI-MS: m/z = 721 (M + H)^+^.

### Biodistribution

**Fig. 4:**
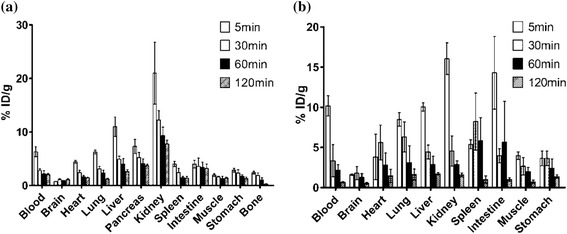
The biodistributions of ^18^F-FP-DPAZn2 (**a**) and ^18^F-FB-DPAZn2 (**b**) in normal mice

The biodistribution of ^18^F-FP-DPAZn2 was evaluated in normal mice, as summarized in Fig. 4. ^18^F-FP-DPAZn2 had the highest uptake in kidney and gradually washout from 20.99 ± 5.77% ID/g to 7.78 ± 0.71% ID/g in the whole process. The radiopharmaceuticals rapidly cleared from live and decreased to a low-level after 120 min post-injection. The pancreas had a high uptake level at 5 min (7.29 ± 1.32% ID/g) and decreased to 3.70 ± 0.26% ID/g after 120 min. But the uptake of brain kept at a relatively low level from 5 min to 120 min post-injection. Bone and muscle also kept at low uptake level of radioactivity during the whole observed time. Other tissues, including intestine, spleen, stomach, lung, blood, and heart, showed moderate uptake of radioactivity in the study process of 2 h.

### PET imaging with ^18^F-FP-DPAZn2

**Fig. 5:**
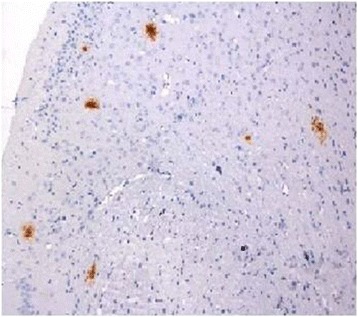
β-amyloid (Aβ) immunohistochemistry of double transgenic AD models

**Fig. 6:**
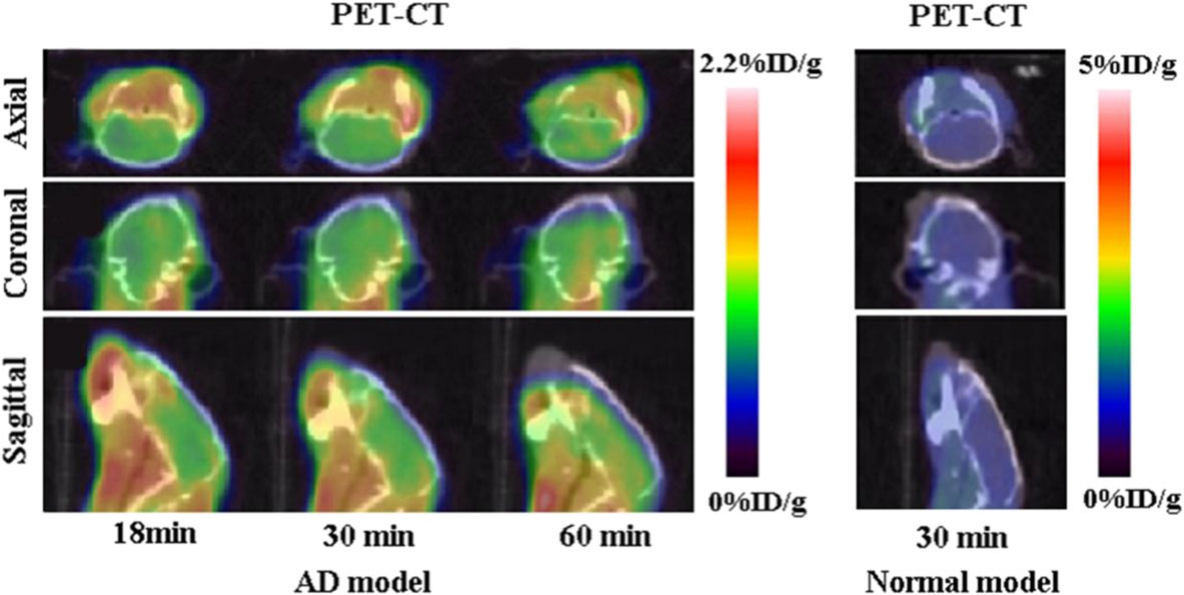
Decay-corrected axial, coronal and sagittal ^18^F-FP-DPAZn2 PET images of AD model mice and normal mice

Depositions of β-amyloid peptide in cerebral tissue of double transgenic AD model were confirmed by immunehisto-chemistry as shown in Fig. 5 [28]. Axial, coronal and sagittal PET images of ^18^F-FP-DPAZn2 obtained in AD model and normal mice are shown in Fig. 6. In normal mice, there was almost no brain uptake of ^18^F-FP-DPAZn2. But in the AD model mice, brain uptake of ^18^F-FP-DPAZn2 was clearly observed at 18 min post-injection, and remained stable accumulation was observed at 30 and 60 min post-injection. Uptake ratio of ^18^F-FP-DPAZn2 in AD brain to normal brain was 1.35 at 18 min, 1.65 at 30 min and 1.88 at 60 min, respectively.

## Discussion

Due to multi-step reaction synthesis, there were not enough reaction vessels and heaters to be used in the PET-MF-2V-IT-I synthesizer. So, we added a vial B10* in the modified synthesizer in order to perform the reaction of ^18^F-FP-DPA with Zn(NO_3_)_2_, which shared the same heater (H_3_) with the vial B10. After this simple improvement, semi-automatic synthesis ^18^F-FP-DPAZn2 was smoothly carried out and the synthesis time reduced by 15 min. Furthermore, the modified method provided a little higher decay-corrected radiochemical yield than the manual operation. The total decay-corrected radiochemical yield of ^18^F-FP-DPAZn2 was 35 ± 6% (*n* = 5) from ^18^F^−^ within 105 ± 10 min, which could give enough radioactivities for the upcoming animal-model PET imaging.

For the labeling of large-molecule weight peptides and proteins, both ^18^F-SFB and ^18^F-NFP as fluoroacylation prosthetic groups appear equally well suited. For the labeling of small-molecule peptides, ^18^F-NFP is to be preferred since it is much smaller steric hindrance than ^18^F-SFB, while the larger ^18^F-SFB will increase the lipophihcity of the labeled compound [29]. Therefore, DPA2 was labeled with ^18^F-SFB in our previous work [20] and labeled with ^18^F-NFP in this study. Compared with ^18^F-FB-DPAZn2, ^18^F-FP-DPAZn2 took a little long whole synthesis time, but gave higher repeated radiochemical yield than ^18^F-FB-DPAZn2 (24 ± 4%).

Fluorescent DPAZn2 probes were shown to have the capacity of selective targeting to apoptosis and necrotic cells [15, 16]. In the previous research, our group reported that ^18^F-labeled DPAZn2 complex ^18^F-FB-DPAZn2 [20] was a potential tracer to evaluate the efficiency of liver cancer treated with chemotherapy. However, ^18^F-FB-DPAZn2 had unfavorable in vivo pharmacokinetics. So, in the current study, ^18^F-FP-DPAZn2 was assessed with potential advantages over ^18^F-FB-DPAZn2. The biodistribution showed that ^18^F-FP-DPAZn2 clearance was mainly through the kidney, as verified by high uptake of kidney at 5 min post-injection and gradual washout after 30 min, and liver was subordination excretion pathway. It has been reported that synthetic small molecular weight imitated Annexin V (Fluorescent DPAZn2) binding quickly to PS-enriched cytomembrane could be an ideal choice [10]. ^18^F-FP-DPAZn2 as synthetic mimic of annexin V had an advantage of smaller molecular weight over ^18^F-FB-DPAZn2. Abdominal uptake of ^18^F-FP-DPAZn2 was also lower than that of ^18^F-FB-DPAZn2, as shown in Fig. 4.

Increasing evidence indicates that small soluble aggregates or oligomers of Aβ_42_, rather than monomers of fibrils, are the most likely neurotoxin in AD [30], which can induce calcium ion influx, calcium ion overload and apoptosis in brain granule cells [31]. It also shows that aging enables Ca^2+^ superload and neural cell death induced by Aβ_42_ oligomers in hippocampal neurons [32]. Mutations of the presenilin genes in the form of amyloid precursor protein possibly result in increased apoptosis of transgenic mice and neural cell culture [33]. Amyloid precursor protein transgenic mice share several critical subcellular alterations with AD, making them valuable models to study mechanisms of neurodegeneration and plaque formation.

In our studies, moderate uptake of ^18^F-FP-DPAZn2 in the AD model was observed during the whole study, while the background was reduced, as shown in Fig. 6. But uptake of ^18^F-FP-DPAZn2 in normal mice was very low. These results were consistent with those of in vivo biodistribution. The comparative results showed that ^18^F-FP-DPAZn2 as an apoptosis agent for AD imaging was possible. We also deduce that ^18^F-FP-DPAZn2 seems to be a promising candidate as a cell death tracer, but which needs to be further investigated.

## Conclusion

By modified commercial PET-MF-2V-IT-I synthesizer, we successfully performed semi-automated production of ^18^F-FP-DPAZn2. The prosthetic group ^18^F-NFP was automatically synthesized from one-pot three-step reaction procedure. ^18^F-FP-DPAZn2 was obtained from the reaction of the precursor DPA2 with ^18^F-NFP, following the reaction of ^18^F-FP-DPA2 with Zn(NO_3_)_2_. The semi-automated radiosynthesis method could afford enough radioactivities and good radiochemical purity of ^18^F-FP-DPAZn2 as a cell death imaging agent for the further in vivo PET imaging study. PET imaging suggested that ^18^F-FP-DPAZn2 could be an effective PET tracer for AD cell apoptosis.

## Abbreviations

^18^F-FP-DPAZn2: *N*-(2-^18^F-fluoropropionyl)-*bis*(*zinc*(II)-dipicolylamine)
DPAZn2: bis(zinc(II)-dipicolylamine)
^18^F-NFP: 4-nitrophenyl-2-^18^F-fluoropropionate
^18^F-SFB: *N*-succinimidyl-4-^18^F-fluorobenzoate
PET: Positron emission tomography
^18^F-FB-DPAZn2: 4-^18^F-fluorobenzoyl-bis(zinc(II)-dipicolylamine
